# Increased replication of CD4^+^ naive T cells and changes in T cell homeostasis in a case of acute exacerbation of juvenile idiopathic arthritis: a case comparison study

**DOI:** 10.1186/1752-1947-7-135

**Published:** 2013-05-21

**Authors:** Giovanni Almanzar, Manuela Zlamy, Christian Koppelstaetter, Andrea Brunner, Verena Jeller, Christina Duftner, Christian Dejaco, Juergen Brunner, Martina Prelog

**Affiliations:** 1 Department of Pediatrics, University of Würzburg, Würzburg, Germany; 2 Department of Pediatrics, Medical University Innsbruck, Innsbruck, Austria; 3 Department of Internal Medicine, Medical University Innsbruck, Innsbruck, Austria; 4 Department of Pathology, Medical University Innsbruck, Innsbruck, Austria; 5 Department of Internal Medicine, Medical University Graz, Graz, Austria

**Keywords:** Exacerbation, Juvenile idiopathic arthritis, Naive T cells, T cell receptor excision circles

## Abstract

**Introduction:**

Juvenile idiopathic arthritis is a heterogeneous T cell-mediated autoimmune disease with symptoms of premature aging of the immune system (immunosenescence). The present work is an investigation of immunosenescence parameters, such as quantity of naive and CD28^-^ T cells, T cell receptor excision circles, relative telomere length and alterations of peripheral T cell replication, and was performed via comparison of a case of acute exacerbation of juvenile idiopathic arthritis against six patients with juvenile idiopathic arthritis with disease remission and six age-matched healthy donors over a follow-up course of 12 months.

**Case presentation:**

Phenotypical T cell characterization and intracellular interferon γ, tumor necrosis factor α, and interleukin 2 production were studied in peripheral blood mononuclear cells from seven patients with juvenile idiopathic arthritis and six healthy control donors, with findings determined by flow cytometry. T cell receptor excision circles and relative telomere length quantification were performed on deoxyribonucleic acid isolated from naive (CD4^+^CD28^+^CD45RA^+^) T cells and investigated via reverse transcription polymerase chain reaction. Ki67 expression was studied by immunohistochemistry on naive T cells. The non-parametric Mann-Whitney U test and Wilcoxon test for two independent groups of variables were used to compare healthy donors with patients with juvenile idiopathic arthritis. During follow-up, patients with juvenile idiopathic arthritis showed lower total counts of naive and CD28-expressing T cells compared to healthy donors. Acute exacerbation led to low naive and CD28^+^ T cell populations and elevated proportions of Ki67-expressing CD4^+^ naive T cells. In conditions of exacerbation, T cell receptor excision circle numbers were in the lower range in patients with juvenile idiopathic arthritis and increased after follow-up. Healthy donors showed significantly higher relative telomere lengths compared to patients with juvenile idiopathic arthritis.

**Conclusions:**

This investigation illustrates that the changes in T cell homeostasis in patients with juvenile idiopathic arthritis may be the result of several mechanisms, such as diminished thymus function and peripheral exertions to maintain the peripheral T cell pool. The results also demonstrate that hallmarks of immunosenescence such as decreased naive T cell levels and lower T cell receptor excision circle numbers can only be interpreted together with replication markers such as relative telomere length or Ki67 expression.

## Introduction

Juvenile idiopathic arthritis (JIA) is a heterogeneous T cell-mediated autoimmune disease. Common to all seven subtypes of JIA, disease onset is prior to the age of 16 years and is characterized by a chronicity of at least six weeks. For adult rheumatoid arthritis (RA), it has been suggested that T cells play an important role in the pathogenesis of the disease. Patients with RA present with immune system abnormalities that resemble the typical characteristics of immune dysfunction described in older patients [1]. Immunological investigations of patients with RA have revealed disturbed T cell homeostasis and diminished thymus output, which was characterized by decreased amounts of T cell receptor excision circles (TRECs) and compensatory peripheral T cell proliferation with telomere shortening [2] and loss of the co-stimulatory molecule CD28, a typical sign of replicatively stressed cells [3]. Patients with JIA demonstrate premature immunosenescence of the CD4^+^ naive T cell pool with age-inappropriate low TREC numbers, shortened telomere lengths and increased peripheral replication of peripheral naive T cells [4]. TRECs are stable deoxyribonucleic acid (DNA) episomes forming during T cell receptor rearrangement. TRECs are not replicated during mitosis and are diluted out during cell divisions. Therefore, TREC counts in naive T cells are not only recognized as a marker for recent thymic emigrants (RTE), but are also influenced by peripheral replication of naive T cells [5]. Telomeres are TTAGGG-rich repeats located at the ends of chromosomes and play an important role in DNA replication and preservation of chromosome integrity. Telomere erosion has been considered a mitotic clock, with the telomere length approximately reflecting the life history of divisions of individual cells [6].

The influence of clinical disease activity on these parameters is still unclear in JIA. The present investigation of immunosenescence parameters was performed via comparison of a case of acute exacerbation of JIA against six patients with JIA with disease remission and six age-matched healthy donors (HD) over a follow-up course of 12 months.

## Case presentation

### Materials and methods

#### Study population

Peripheral blood mononuclear cells (PBMCs) were obtained from seven patients with JIA (all girls), specifically with extended oligoarticular JIA. All patients were tested and found to be rheumatoid factor (RF) negative and anti-nuclear antibody (ANA) positive, and fulfilled the International League of Associations for Rheumatology (ILAR) classification for JIA [7]. PMBCs were also obtained from six age-matched and sex-matched healthy donors (HD) (Table 1). All donors were recruited at the Department of Pediatrics, Medical University Innsbruck.

**Table 1:** Matching of the juvenile idiopathic arthritis (JIA) group (patients 1 to 6), our patient with JIA with acute exacerbation (patient 7), and healthy donors (patients 8 to 13)

**Patient**	**Age (months)**	**Disease onset (months)**	**Disease duration (months)**
1	191	44	147
2	143	44	99
3	161	37	124
4	157	61	96
5	196	42	154
6	173	49	124
7	192	48	144
8	184	-	-
9	136	-	-
10	156	-	-
11	144	-	-
12	204	-	-
13	172	-	-

One of the patients with JIA was a 16-year-old girl (patient 7 in Table 1), with extended oligoarticular JIA (ANA positive test result, 1:320; RF negative test result) with disease onset at three years of age. Our patient presented with acute disease exacerbation at the time of evaluation. She was classified as in clinical remission off medication for over 12 months, as according to published criteria for disease remission [8]. Disease exacerbation at the time of evaluation was verified by clinical examination (extended oligoarthritis with painful joints of the upper and lower extremities) and increased laboratory parameters (C-reactive protein level [CRP] 5.14mg/dL; erythrocyte sedimentation rate [ESR] 99mm/first hour, 152mm/second hour; ANA 1:640). After acute exacerbation, our patient was treated with indometacin (2mg/kg/day) and prednisolone (1mg/kg/day) for four weeks. Methotrexate was administered (10mg/m^2^ body surface/week) orally. Over a six-month period, our patient showed disease remission on methotrexate and prednisolone (<0.1mg/kg/day) treatment (that is, remission on medication) [8]. A 12-month follow-up sample was taken under conditions of disease remission off medication for at least five months.

Patients 1 to 6 (see Table 1) were in clinical remission off medication for at least 12 months [8]. The criteria for disease remission included the following: no active arthritis, no fever, no rash, serositis, splenomegaly, or generalized lymphadenopathy attributable to JIA, no active uveitis, normal ESR or CRP level, and a physician global assessment of disease activity indicating clinical disease quiescence. After 12 months of follow-up, patients 1 to 6 were still in remission off medication [8].

Controls were healthy according to World Health Organization (WHO) definition. None of the HD had a personal or family history of inflammatory disease or received medications. Unfortunately, HD blood samples were not collected after 12 months because donors refused to have samples taken. However, significant changes to the investigated immunosenescence parameters were not expected in healthy young individuals during this period of time. Therefore, recruitment of the original HD group did not appear to be mandatory to fulfill the scope of the study and the missing follow-up data for HD were neglected. The study was performed according to the Declaration of Helsinki (2000) and approved by the local ethics committee (Medical University Innsbruck). All patients with JIA and HD gave their written informed consent to participate in this study.

#### Separation of T cell subsets

PBMCs were isolated by using LymphoPrep™ (Axis Shield, Oslo, Norway) according to the manufacturer’s instructions. CD4^+^CD28^+^CD45RA^+^ (naive) T cells were separated by positive selection using CD4, CD45RA, CD28 antibodies labeled with magnetic beads and an autoMACS™ with sterile columns (Miltenyi Biotec, Teterow, Germany). The purity of separated CD4^+^CD28^+^CD45RA^+^ T cells was checked using four-color flow cytometry (FACSCalibur™ flow cytometer; Becton Dickinson, Oxford, UK) and ranged from 97 percent to 99 percent.

#### Quantification of T cell subsets

PBMCs were incubated with monoclonal mouse antibodies (mAbs) specific for CD3, human leukocyte antigen (HLA)-DR, CD4, CD8, CD28, CD25, CD45RA, CD45RO, and CD62L labeled with fluorescein isothiocyanate (FITC), phycoerythrin (PE), peridinin-chlorophyll-protein complex (PerCP) or allophycocyanin (APC) (all antibodies were purchased from BD Pharmingen™, San Jose, CA, USA) for 20 minutes at room temperature in the dark. After incubation, red blood cell lysis was performed with FACS™ Lysing Solution (BD Pharmingen™, San Jose, CA, USA). Subsequently, cells were washed twice with phosphate-buffered saline and fixed with 2 percent paraformaldehyde. All analyses were performed using a FACSCalibur™ flow cytometer utilizing CellQuest™ software (DB Pharmingen™, San Jose, CA, USA). According to phenotypic CD markers, CD28^+^CD45RA^+^ and CD45RA^+^CD62L^+^ T cells were characterized as naive, CD45RO^+^ as memory T cells and CD4^+^CD25^+^CD62L^+^ as regulatory T cells.

#### Quantification of TREC numbers

TREC numbers within CD4^+^CD28^+^CD45RA^+^ T cells were assessed. DNA was extracted from separated CD4^+^CD28^+^CD45RA^+^ T cells using QIAamp® DNA Mini Kit (Qiagen, Chatsworth, CA, USA). Signal-joint TREC concentrations were determined by quantitative SYBR® Green real-time polymerase chain reaction (PCR) based on the coding TREC sequence using an iCycler iQ™ Real-Time Quantitative PCR Detection System (Bio-Rad Laboratories, Hercules, Canada). We designed primers to amplify a DNA fragment of 82bp across the remaining recombination sequence δrec/ψalpha (5’-CACATCCCTTTCAACCATGCT-3’ and 5’-GCCAGCTGCAGGGTTTAGG-3’). For quantification we used the internal standard as previously described [9]. To avoid bias by different numbers of naive T cells, TRECs were calculated in relation to CD4^+^CD28^+^CD45RA^+^ T cell numbers.

#### Telomere length analysis

Determination of relative telomere length (RTL) was performed in separated CD4^+^CD28^+^CD45RA^+^ T cells by calculating the ratio of a quantitative PCR reaction product from the same sample using specific primers for telomeres and a single copy gene. Quantitative PCR is the method of choice for determining telomere lengths in small extractable quantities of DNA, as was the case in our study.

#### Ki67 staining

Naive T cells in the cell cycle were identified by expression of the Ki67 nuclear antigen. Determination of Ki67 expression on CD4^+^CD28^+^CD45RA^+^ T cells was performed by cytospin preparation (Shandon Cytospin® 4, Waltham, MA, USA) of 5×10^3^ CD4^+^CD28^+^CD45RA^+^ T cells and permeabilized with TRITON™ X-100 (Sigma-Aldrich, St Louis, MO, USA). Immunohistochemistry was undertaken in a fully automated NexES® IHC system (Ventana Medical Systems, AZ, USA). For microscopic orientation counterstaining was performed with Hematoxylin Counterstain and Bluing Reagent (Ventana Medical Systems, AZ, USA). Stained sections were examined by two independent investigators (MP and AB) by light microscopy (Eclipse 800, Nikon, Japan). Percentages of Ki67^+^ cells were calculated per total CD4^+^CD28^+^CD45RA^+^ T cell counts on each cytospin preparation.

#### Intracellular cytokine production

For intracellular staining, cells were stimulated with 25ng/mL phorbol 12-myristate 13-acetate and 1μg/mL ionomycin in the presence of 10μg/mL brefeldin A for four hours (Sigma-Aldrich, Munich, Germany). After cell surface staining for CD4, CD8 and CD45RA with subsequent fixation and permeabilization, cells were stained with FITC-conjugated anti-interferon (IFN)γ, anti-tumor necrosis factor (TNF)α, and anti-interleukin (IL)-2, antibodies or control immunoglobulin (R&D Systems Inc., Minneapolis, MN, USA). For intracellular cytokine staining, additional markers for naive T cells, such as CD28, could not be implemented because of technical limitations. Final fixation was achieved using 1 percent cell fix (Becton Dickinson, San Diego, CA, USA). Data were analyzed using WinMDI software (V. 2.8, Joseph Trotter, Scripps Research Institute, La Jolla, CA, USA).

#### Statistical analysis

The non-parametric Mann-Whitney U test for two independent groups of variables was used to compare HD with patients with JIA (SPSS V.15.0; SPSS Inc., Chicago, IL, USA). The non-parametric Wilcoxon test for pair differences was used for two dependent groups of variables (JIA and JIA follow-up). A p-value of <0.05 was considered significant.

### Results

#### Lower naive T cell counts in patients with JIA

**Figure 1:**
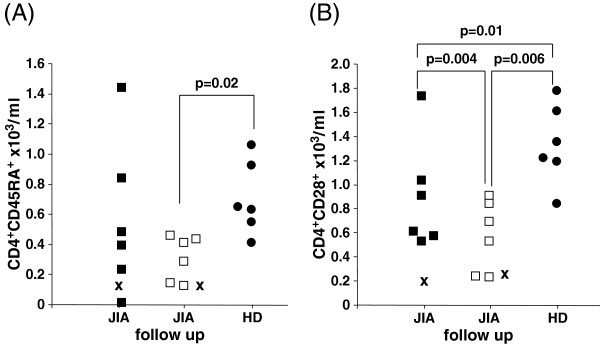
**Lower naive T cell counts in patients with Juvenile Idiopathic Arthritis (JIA).** Lower CD4^+^CD28^+^CD45RA^+^ (**A**) and CD4^+^CD28^+^ T cell counts (**B**) in patients with JIA are shown. Closed squares show patients with juvenile idiopathic arthritis (JIA), open squares JIA follow-up after 12 months, X indicates our patient with JIA with acute exacerbation, and closed circles show healthy donors (HD). A p-value of <0.05 was considered significant.

CD45 isoforms and CD28 surface marker expression were used to define naive and memory T cells in peripheral CD4^+^ and CD8^+^ subsets in our patient with acute exacerbation, patients with JIA in remission at the beginning and over a 12-month follow-up study and age-matched HD (Tables 1 and 2). Lack of CD28 expression on both CD4^+^ and CD8^+^ T cells has been associated as a biological indicator of aging of the immune system upon continuous antigen stimulation and subclinical inflammation [10] and was therefore included in the study. There was a significantly lower number of CD4^+^CD28^+^CD45RA^+^ T cells (P=0.02) and CD8^+^ CD28^+^CD45RA^+^ T cells (P=0.04) (Table 2) in the follow-up evaluation of patients with JIA compared with HD (Figure 1A, Table 2). Patients with JIA showed significantly lower numbers of CD4^+^CD28-expressing cells as compared with HD (P=0.01), and the difference was more pronounced after 12 months (P=0.004) (Figure 1B). The same effect was also observed in CD8^+^CD28-expressing cells (P=0.02) (Table 2). In the acute exacerbation stage in our patient (patient 7), this effect was more dramatic. Our patient showed low numbers of CD45RA-expressing and CD28-expressing T cells at the beginning and at follow-up (Figure 1A,B), and also showed lower percentages of CD28^+^ T cells within the CD4^+^ T cell pool at acute exacerbation (27.8 percent) and at follow-up (33.3 percent) compared to patients with JIA (mean 95.7 percent; at follow-up 96.6 percent) and HD (96.9 percent).

**Table 2:** T cell subset counts in patients with juvenile idiopathic arthritis (JIA) and healthy donors (HD)

	**Patient with JIA with acute exacerbation/follow-up**	**JIA group (n=6)**	**JIA follow-up (n=6)**	**HD (n=6)**
CD3^+^	1.1/1.3	1.73±0.68 (1.60; 1.10 to 2.80)	1.04±0.49 (0.98; 0.51 to 1.72)^++^	2.18±0.36 (2.0; 1.90 to 2.70)
CD3^+^HLA-DR^+^	0.06/0.03	0.09±0.01 (0.09; 0.08 to 0.11)	0.09±0.05 (0.11; 0.03 to 0.16)	0.13±0.03 (0.12; 0.11 to 0.19)^xx^
CD3^+^CD4^+^	0.72/0.93	0.94±0.48 (0.79; 0.55 to 1.83)	0.59±0.31 (0.62; 0.24 to 0.96)^++^	1.31±0.35 (1.24; 0.88 to 1.84)
CD4^+^CD25^+^CD62L^+^	0.12/0.04	0.19±0.18 (0.15; 0.03 to 0.52)	0.14±0.09 (0.13; 0.05 to 0.26)	0.25±0.08 (0.23; 0.15 to 0.35)
CD4^+^CD28^+^	0.20/0.31	0.90±0.46 (0.76; 0.53 to 1.73)***	0.57±0.29 (0.62; 0.23 to 0.90)^+++^	1.27±0.34 (1.22; 0.84 to 1.78)^xx^
CD4^+^CD28^+^CD45RA^+^	0.15/0.16	0.33±0.24 (0.25; 0.001 to 0.62)	0.31±0.15 (0.35; 0.13 to 0.46)^++^	0.49±0.012 (0.45; 0.34 to 0.63)^xx^
CD4^+^CD45RA^+^CD62L^+^	0.22/0.04	0.39±0.24 (0.43; 0.001 to 1.14)	0.19±0.08 (0.19; 0.09 to 0.28)	0.34±0.32 (0.16, 0.19 to 0.61)
CD4^+^CD45RO^+^	0.12/0.15	0.08±0.07 (0.06; 0.01 to 0.22)	0.22±0.13 (0.23; 0.07 to 0.38)	0.15±0.11 (0.17; 0.001 to 0.29)
CD3^+^CD8^+^	0.34/0.37	0.50±0.19 (0.49; 0.27 to 0.73)	0.41±0.18 (0.37; 0.21 to 0.65)^++^	0.78±0.14 (0.76; 0.63 to 0.96)
CD8^+^CD28^+^	0.28/0.32	0.41±0.16 (0.41; 0.18 to 0.64)	0.31±0.15 (0.26; 0.17 to 0.51)^++^	0.61±0.11 (0.64; 0.47 to 0.75)
CD8^+^CD28^+^CD45RA^+^	0.30/0.25	0.57±0.51 (0.43; 0.01 to 1.44)	0.29±0.13 (0.27; 0.16 to 0.47)^+^	0.66±0.24 (0.63; 0.41 to 1.06)
CD8^+^CD45RA^+^CD62L^+^	0.07/0.09	0.31±0.25 (0.20; 0.05 to 0.53)***	0.19±0.07 (0.16; 0.12 to 0.29)	0.32±0.29 (0.13; 0.19 to 0.52)
CD8^+^CD45RO^+^	0.38/0.90	0.23±0.12 (0.23; 0.03 to 0.41)**	0.70±0.30 (0.61; 0.30 to 1.13)	0.41±0.25 (0.42; 0.001 to 0.69)

Total counts ×10^3^ cells/μL are given as mean±standard deviation (median; range). Significant differences between patients with JIA (JIA group) and JIA follow-up are indicated with **p<0.025, ***p<0.01. Significant differences between JIA follow-up and HD are indicated with ^+^p<0.05, ^++^p<0.025, ^+++^p<0.01. Significant differences between JIA group and HD are indicated with ^xx^p<0.025.

#### Increased replication of CD4^+^ naive T cells in acute exacerbation of JIA

Reduction of TRECs is a function of both thymic output of RTE and of replication of peripheral naive T cells [6]. Therefore, TREC numbers should be interpreted in correlation with replicative markers, such as telomere length or Ki67 expression. Proliferative status, relative telomere length (RTL), and TRECs were assessed in peripheral blood samples from our patient with acute exacerbation, patients with JIA, and HD.

**Figure 2:**
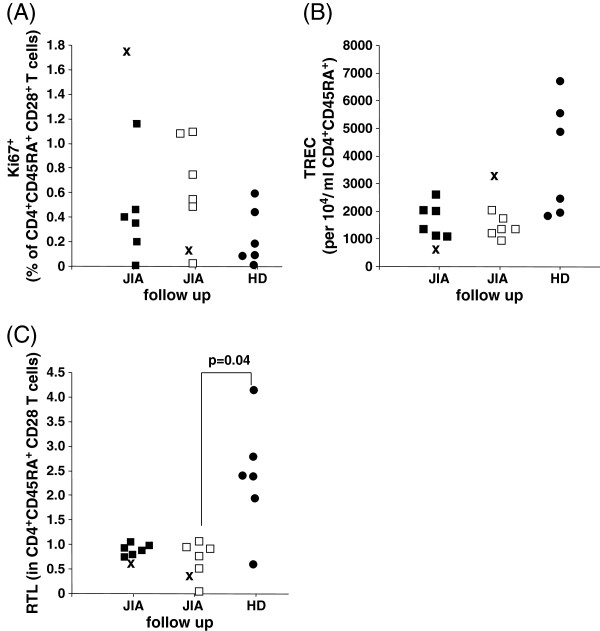
**Proliferative measures in Juvenile Idiopathic Arthritis (JIA) patients.** Ki67-expressing CD4^+^CD28^+^CD45RA^+^ T cells (**A**), T cell receptor excision circles (TRECs) (**B**), and relative telomere length (RTL) (**C**). Closed squares show patients with juvenile idiopathic arthritis (JIA), open squares JIA follow-up after 12 months, X indicates our patient with JIA with acute exacerbation, and closed circles show healthy donors (HD). A p-value of <0.05 was considered significant.

An increased proportion of Ki67-expressing CD4^+^CD28^+^CD45RA^+^ naive T cells (1.68 percent) and a relatively low proportion of replicating cells after remission (0.15 percent) was determined in our patient with acute exacerbation (Figure 2A). In contrast, in patients with JIA (patients 1 to 6 in Table 1), the proportion of replicating cells (Ki67^+^) increased after 12 months (0.43±0.40 percent to 0.66±0.41 percent after follow-up) (Figure 2A). Relative low Ki67 expression (0.23±0.19 percent; not significant) was detected in HD (Figure 2A).

With regard to TREC numbers, no changes were observed in patients with JIA in remission at first evaluation (1649±586 per 10^4^ CD4^+^CD28^+^CD45RA^+^ T cells) or after 12 months (1424±384 per 10^4^ CD4^+^CD28^+^CD45RA^+^ T cells) (Figure 2B), while TREC number dramatically increased in our patient with acute exacerbation after 12 months (exacerbation: 737 per 10^4^ CD4^+^CD28^+^CD45RA^+^ T cells; follow-up: 3749 per 10^4^ CD4^+^CD28^+^CD45RA^+^ T cells) (Figure 2B). Slightly higher TREC numbers were detected in naive T cells (3685±2264 per 10^4^ CD4^+^CD28^+^CD45RA^+^ T cells) in HD (Figure 2B). Moreover, our patient (patient 7 in Table 1) during exacerbation and after 12 months showed lower RTL (during exacerbation: 0.67; follow-up: 0.37) compared to patients with JIA (patients 1 to 6 in Table 1) in remission (0.90±0.12; JIA follow-up: 0.69±0.38) (Figure 2C). Significantly higher RTL were determined in HD (2.45±1.27; P=0.04) (Figure 2C).

#### Disturbed cytokine production in our patient with acute exacerbation of JIA

TNFα, IFNγ, and IL-2 production was determined in CD45RA-expressing and CD45RO-expressing CD4^+^ and CD8^+^ T cells at acute exacerbation of our patient by intracellular cytokine analysis. No significant differences were determined in the cytokine production in the four T cell subpopulations in patients with JIA or HD (Table 3). However, a higher proportion of IL-2 producing CD45RA^+^ and CD45RO^+^ T cells were detected in patients with JIA compared with the HD group (Table 3). Remarkable increased proportions of TNFα, IFNγ and IL-2 producing CD45RO^+^ T cells were determined in our patient with acute exacerbation (Table 3).

**Table 3:** Intracellular cytokine production during acute exacerbation

	**Patient with JIA with acute exacerbation**	**JIA group (n=6)**	**HD group (n=6)**
TNFα (percentage of CD4^+^CD45RA^+^)	6.09	6.99±2.52 (6.37; 4.62 to 10.43)	6.26±4.60 (4.47; 1.82 to 12.9)
IFNα (percentage of CD4^+^CD45RA^+^)	19.63*	2.77±1.24 (2.60; 1.15 to 4.39)	4.04±2.21 (3.52; 1.39 to 7.49)
IL-2 (percentage of CD4^+^CD45RA^+^)	53.74	37.63±31.49 (28.65; 5.53 to 81.25)	14.68±9.79 (14.54; 3.12 to 28.56)
TNFα (percentage of CD4^+^CD45RO^+^)	41.99*	21.11±9.17 (21.18; 7.74 to 34.36)	18.19±8.79 (15.38; 11.31 to 34.69)
IFNα (percentage of CD4^+^CD45RO^+^)	41.23*	10.05±4.09 (10.12; 4.52 to 14.46)	13.30±6.07 (12.08; 8.21 to 24.21)
IL-2 (percentage of CD4^+^CD45RO^+^)	79.77	50.09±30.69 (41.09; 15.65 to 90.03)	31.01±22.65 (24.37; 8.56 to 60.41)
TNFα (percentage of CD8^+^CD45RA^+^)	8.19	7.05±3.64 (6.40; 2.70 to 21.30)	5.39±4.04 (4.35; 1.44 to 11.80)
IFNγ (percentage of CD8^+^CD45RA^+^)	18.21	11.27±8.12 (8.19; 4.49 to 24.05)	8.68±5.13 (6.86; 4.61 to 18.20)
IL-2 (percentage of CD8^+^CD45RA^+^)	54.61*	25.73±26.00 (16.35; 6.63 to 17.52)	12.49±9.45 (8.44; 3.99 to 25.89)
TNFα (percentage of CD8^+^CD45RO^+^)	68.22*	24.04±10.02 (27.43; 11.44 to 33.10)	22.98±9.35 (22.6; 12.27 to 35.64)
IFNγ (percentage of CD8^+^CD45RO^+^)	83.67*	28.26±19.57 (27.43; 2.63 to 50.26)	34.77±17.44 (33.33; 9.14 to 61.50)
IL-2 (percentage of CD8^+^CD45RO^+^)	73.48*	34.82±15.52 (31.53; 17.52 to 58.08)	23.84±22.63 (12.86; 4.58 to 60.27)

Total counts ×10^3^ cells/μL are given as mean±standard deviation (median; range). Values from our patient with JIA with acute exacerbation (patient 7) below or above the range found in the JIA group are indicated with *.

HD, healthy donors; IFN, interferon; IL, interleukin; JIA, juvenile idiopathic arthritis; TNF, tumor necrosis factor.

## Discussion

Our patient’s case gives an impression of disturbed peripheral CD4^+^CD28^+^CD45RA^+^ T cell homeostasis under the condition of acute disease exacerbation and underlines the importance of considering disease activity when evaluating immunological aging markers. Although limited by a small number of patients with JIA, the present study corroborates the fact that markers of immunosenescence such as decreased naive T cells and lower TREC numbers can only be interpreted correctly together with replication markers such as telomere length or Ki67 expression. Intracellular cytokines may be supportive parameters but are shown to be too variable to act as reliable markers to study clinical aspects of disease activity.

Loss of CD28 expression has been suggested as a marker of replicative senescence for T cells [10]. A clonal expansion of CD8^+^CD28^-^ T cells was found in older people [10] and individuals with RA [3]. The data from the follow-up cohort confirm the findings of an accelerated decrease of naive T cells in patients with JIA with advancing chronological age [4], which was also demonstrated for patients with RA [1,2]. However, this comparison is limited as we were not able to provide follow-up data of the HD group. Although acute exacerbation did not change the total counts of CD4^+^CD28^+^CD45RA^+^ or total CD28-expressing T cells, percentages of CD28-expressing CD4^+^ T cells were significantly decreased in our patient with acute exacerbation, whereas percentages of CD28-expressing CD4^+^ T cells were different between JIA follow-up and HD.

TRECs were evaluated to estimate thymic output and peripheral T cell turnover under the condition of acute disease exacerbation. TREC numbers were lower in patients with JIA compared to HD and unaltered by follow-up in the JIA group. These results corroborate the findings that patients with JIA show age-inappropriate lower TREC numbers but with no correlation with chronological age [4]. Our patient with acute exacerbation showed relatively low TREC numbers during the acute exacerbation period compared to patients with JIA with disease remission. In our patient with acute exacerbation, enhanced TREC numbers and even higher TREC numbers than the other patients with JIA were found after disease remission, suggesting recovery of thymus function after successful treatment. This is in accordance with findings in children and younger adults with acquired immunodeficiency syndrome (AIDS) under highly active anti-retroviral therapy (HAART) [11] in whom recovery to higher levels than before HAART was found. The study suggested that ongoing production of naive CD4^+^ T cells in the thymus is most active during the earlier stages of HAART and declines later during HAART. However the precise timing of when children show maximum thymic output during HAART remains unclear. An increase in the fraction of TREC^+^ T cells per naive T cell, which was equivalent to a faster growth of TREC^+^ T cells versus total naive T cells was also found for CD4^+^ T cells following initiation of HAART [12]. When discussing the TREC dynamics in our patient with acute exacerbation, we cannot sufficiently answer the question as to whether our patient already had low TREC numbers before exacerbation, as found in children with AIDS before initiation of HAART.

However, TREC numbers are also influenced by peripheral proliferation and, therefore, dilution of TRECs may be caused by increased replication of peripheral CD4^+^ naive T cells, which may be confirmed by enhanced Ki67-expressing T cells during acute exacerbation in our patient. Additionally, decreased thymic output will induce compensatory autoproliferation, a process that contributes to TREC dilution. Chronic T cell stimulation induces increased T cell turnover in patients with JIA, as demonstrated by age-inappropriate short T cell telomere lengths in patients with JIA. Age-inappropriate thymic involution and increased peripheral T cell turnover may each contribute to the lower TREC numbers observed in patients with JIA. These data highlight the pitfalls of interpretation of TREC data [13], therefore, replication markers such as Ki67 expression and RTL were considered in our study. TREC numbers were calculated to CD4^+^ naive T cell counts, the latter unaltered by acute exacerbation. In view of these results it has to be considered that the peripheral naive T cell pool consists of two subsets of cells, namely RTE and peripheral replicating cells. Increased replication of naive T cells may be the crucial factor in acute exacerbation and disease activity. However, despite high replication activity increased amounts of naive T cells were not found in the peripheral blood samples from our patient with acute exacerbation, possibly due to accumulation in other compartments, (for example, the inflamed joints). Maturation of naive T cells to CD45RO^+^ memory T cells may be a possible explanation for disappearance of peripheral blood naive T cells despite increased replicative activity. This was also assumed for a model describing naive T cell dynamics in children and younger adults receiving HAART [12]. Increased priming into memory T cells may be also true for our patient with acute exacerbation, who showed markedly increased CD8^+^CD45RO^+^ counts. During remission, enhanced thymus output of RTE, decreased replication activity of naive T cells, priming into memory T cells and/or depletion of peripheral blood naive T cells by accumulation in other sites may have contributed to the increased TREC numbers in naive T cells seen in the 12-month follow-up of our patient.

There was a tendency to lower RTL in the 12-month follow-up of patients with JIA compared to HD. Although limited by missing follow-up data from HD, these results fit with the tendency of increased Ki67-expressing CD4^+^ naive T cells in patients with JIA compared to HD [4]. Telomeric erosion was also clearly accelerated in patients with RA [2], which was explained as a consequence of compensatory cell renewal.

Cytokines have been implicated in driving homeostatic T cell replication. Their most likely disease-related production could enhance T cell proliferation and exhaust the replicative potential [14]. JIA has been associated with high serum or intra-articular levels of proinflammatory cytokines such as IL-6, IL-1α, IL-1β or TNFα [15]. In fact, our study demonstrated an increase in the levels of TNFα, IFNγ and IL-2 in both CD45RA^+^ and CD45RO^+^, CD4/CD8 T cell subpopulations in patients with JIA. Moreover, high levels of these pro-inflammatory cytokines were observed in our patient with acute exacerbation during the exacerbation state, suggesting a Th1 cytokine-driven mechanism supported by an accumulation of CD45RO^+^ memory T cells and the reduction in the naive T cell population. Interestingly, classical cytokines for memory and effector T cell responses, such as TNFα and IFNγ, were increased in the CD45RA^+^ T cells. One may ask the question whether CD45RA^+^ alone is a reliable marker for naive T cells, as effector T cells also express CD45RA but lose CD28 surface expression. For this reason it may be more likely that the proportions of increased TNFα and IFNγ within the CD45RA^+^ T cell pool are predominately caused by activation of effector T cells.

## Conclusions

Our patient’s case supports the notion that changes in T cell homeostasis in JIA are down to the effect of several mechanisms such as diminished thymus function and peripheral exertions to maintain the peripheral T cell pool [4]. The findings also demonstrate that quantitative T cell measurements or TRECs alone are insufficient to explain the immunological process in acute exacerbation of the disease or in remission. The results should only be interpreted together with data on replication. As, for the most part, the interpretation of the alterations found in our patient’s case remains speculative, there is increased demand for a prospective evaluation of the influence of disease activity as well as of therapy on thymus function and reconstitution of the naive T cell immune system in JIA.

## Consent

Written informed consent was obtained from the patients for publication of this report and accompanying images. A copy of the written consent is available for review by the Editor-in-Chief of this journal.

## Competing interests

The authors declare that they have no competing interests.

## Authors’ contributions

GA performed the flow cytometry analysis and interpretation of data. MZ recruited patients and handled clinical data. CK performed the telomere length analysis and interpretation of telomere data. AB performed the Ki67 analysis and interpretation of proliferation data. VJ performed the lymphocyte isolation, autoMACS™ separation and DNA preparation. CDu performed the analysis of intracellular cytokines by flow cytometry. CDe analysed and interpreted the intracellular cytokine data. JB recruited patients and helped with interpretation of clinical data. MP designed the study and wrote the paper. All authors read and approved the final manuscript.
